# Coadministration of *Hedera helix* L. Extract Enabled Mice to Overcome Insufficient Protection against Influenza A/PR/8 Virus Infection under Suboptimal Treatment with Oseltamivir

**DOI:** 10.1371/journal.pone.0131089

**Published:** 2015-06-22

**Authors:** Eun-Hye Hong, Jae-Hyoung Song, Aeri Shim, Bo-Ra Lee, Bo-Eun Kwon, Hyuk-Hwan Song, Yeon-Jeong Kim, Sun-Young Chang, Hyeon Gun Jeong, Jong Geal Kim, Sang-Uk Seo, HyunPyo Kim, YongSoo Kwon, Hyun-Jeong Ko

**Affiliations:** 1 Laboratory of Microbiology and Immunology, College of Pharmacy, Kangwon National University, Chuncheon, South Korea; 2 Natural Medicine Research Center, Korea Research Institute Bioscience and Biotechnology, Chungcheongbuk-do, Korea; 3 College of Pharmacy, Inje University, Gimhae, South Korea; 4 College of Pharmacy, Ajou University, Suwon, South Korea; 5 Ahngook pharm., R&D Center, Seoul, Korea; 6 Mucosal Immunology Section, International Vaccine Institute, Seoul, South Korea; Seoul National University College of Pharmacy, KOREA, REPUBLIC OF

## Abstract

Several anti-influenza drugs that reduce disease manifestation exist, and although these drugs provide clinical benefits in infected patients, their efficacy is limited by the emergence of drug-resistant influenza viruses. In the current study, we assessed the therapeutic strategy of enhancing the antiviral efficacy of an existing neuraminidase inhibitor, oseltamivir, by coadministering with the leaf extract from *Hedera helix* L, commonly known as ivy. Ivy extract has anti-inflammatory, antibacterial, antifungal, and antihelminthic properties. In the present study, we investigated its potential antiviral properties against influenza A/PR/8 (PR8) virus in a mouse model with suboptimal oseltamivir that mimics a poor clinical response to antiviral drug treatment. Suboptimal oseltamivir resulted in insufficient protection against PR8 infection. Oral administration of ivy extract with suboptimal oseltamivir increased the antiviral activity of oseltamivir. Ivy extract and its compounds, particularly hedrasaponin F, significantly reduced the cytopathic effect in PR8-infected A549 cells in the presence of oseltamivir. Compared with oseltamivir treatment alone, coadministration of the fraction of ivy extract that contained the highest proportion of hedrasaponin F with oseltamivir decreased pulmonary inflammation in PR8-infected mice. Inflammatory cytokines and chemokines, including tumor necrosis factor-alpha and chemokine (C-C motif) ligand 2, were reduced by treatment with oseltamivir and the fraction of ivy extract. Analysis of inflammatory cell infiltration in the bronchial alveolar of PR8-infected mice revealed that CD11b^+^Ly6G^+^ and CD11b^+^Ly6C^int^ cells were recruited after virus infection; coadministration of the ivy extract fraction with oseltamivir reduced infiltration of these inflammatory cells. In a model of suboptimal oseltamivir treatment, coadministration of ivy extract fraction that includes hedrasaponin F increased protection against PR8 infection that could be explained by its antiviral and anti-inflammatory activities.

## Introduction

Influenza viruses are negative-sense RNA viruses of the family *Orthomyxoviridae* [1], and are classified into 3 serotypes, namely A, B, and C. Type A viruses are further classified according to the antigenic variation of surface glycoproteins, hemagglutinin and neuraminidase [2]. Airborne influenza A virus causes severe respiratory disease, and may lead to a pandemic outbreak if spread rapidly [3]. Although influenza infection can be prevented by developing seasonal flu vaccinations based on predicting strains that are likely to circulate in the coming season, it is impossible to be precise.

Anti-influenza drugs reduce fever and other manifestations of the disease induced by influenza virus. M2 proton channel inhibitors, including rimantadine and amantadine, inhibit influenza A virus by blocking the uncoating process [4]. However, influenza viruses resistant to M2 inhibitors are widespread and limit the efficacy of anti-influenza drugs. Oseltamivir, zanamivir, and peramivir are potent and selective inhibitors of neuraminidase proteins found in influenza A and B viruses that were recently introduced to treat infected patients [5,6]. Neuraminidase inhibitors effectively limit influenza virus infection by inhibiting the cleavage of sialic acid residues on newly formed virions, thereby blocking the release and spread of progeny virions [7]. The recent emergence of oseltamivir-resistant influenza virus variants aroused concern about a potential pandemic outbreak [8]. Zanamivir is the only available treatment against oseltamivir-resistant influenza virus [9]; however, zanimivir-resistant mutant virus have been reported, even in the absence of exposure to zanamivir [10]. Furthermore, zanamivir-resistant variants demonstrate cross-resistance to peramivir [10]. The emergence of influenza virus resistant to neuraminidase inhibitors is a matter of great concern, and developing novel anti-influenza drugs with mechanisms of actions independent of neuraminidase should be urgently addressed [11].

*Hedera helix* L., also known as common ivy, is a woody plant belonging to the *Araliaceae* family. Although it is widely known to cause allergic contact dermatitis, ivy leaf extract has been used to treat inflammatory bronchial diseases [12]. Several compounds isolated from ivy, including α-hederin, hederasaponin-C, hederacolchiside-E, and hederacolchiside-F possess anti-inflammatory properties [13]. In the past, the leaves and fruit were used in Europe to treat diseases of the gastrointestinal tract [14]. *H*. *helix* L. was also used for treat inflammation, burns, cough, neuralgia, and rheumatism. Ivy extract may possess antibacterial, antihelmintic, leishmanicidic, and antifungal properties [13]; however, antiviral activity, particularly against influenza virus, has not been reported.

The aims of this study were to develop novel approaches to enhance the antiviral properties of oseltamivir. Influenza A/PR/8 virus (PR8)-infected mice were treated with a suboptimal dose of oseltamivir which leads to insufficient protection against PR8 virus infection. However, coadministration of ivy extract with a suboptimal oseltamivir dose resulted in increased protection of PR8 virus–infected mice, suggesting that ivy extract enables mice to overcome PR8 virus infection in circumstances where oseltamivir efficacy is suboptimal. In vitro cell viability assays using A549 cells infected with PR8 virus indicated that hederasaponin F (HSF), a component of ivy extract, has synergistic anti-PR8 virus activity when combined with oseltamivir treatment. Furthermore, the fraction of ivy extract that contained HSF showed significant anti-influenza activity with reduced pulmonary inflammation when administered with a suboptimal dose of oseltamivir compared with oseltamivir treatment alone. Together, we propose a novel combined anti-influenza therapy consisting of oseltamivir with the HSF-containing fraction of ivy extract. This combination demonstrates significant benefits over oseltamivir treatment alone. It reduces lung inflammation at the latter stage of PR8 virus infection, and has the potential to remove residual influenza virus through the direct antiviral actions of HSF after insufficient oseltamivir treatment.

## Materials and Methods

### Animal preparation

C57BL/6 mice between 6 and 7 weeks of age were purchased from KOATCH Bio (Pyeongtaek, Korea) and maintained in animal facility at the Kangwon National University. The protocol was approved by the Institutional Animal Care and Use Committee of the Kangwon National University (Permit Number: KW-140811-2). Mice (n = 5/group) were intranasally administered with 30 μl of 5 x 10^3^ or 1 x 10^5^ pfu/mouse of PR8 virus for *in vivo* experiments. For lethal dose, we inoculated with 30 μl of 1 x 10^5^ pfu/mouse of PR8 virus to monitor survival of mice. We humanely sacrificed the mice when body weight was reduced as less than 70% of initial weight as permitted by KIACUC, and daily checked the body weight of mice every morning until the end of experiments. For sublethal dose, we administered mice with 30 μl of 5 x 10^3^ pfu/mouse of PR8 virus and also checked the body weight as same way. In sublethal dose, no mice lost their weight less than 70% of initial weight. Virus titration was performed as previously reported [15].

To determine the suboptimal dose of oseltamivir that caused 50% of mice infected with PR8 virus to survive, mice received once-daily oral administration of 1, 5, or 25 mg/kg oseltamivir for 5 consecutive days. To determine the potential anti-influenza virus effect of ivy extract, PR8-infected mice received either 30 mg/kg of ivy extract or vehicle (phosphate-buffered saline [PBS]) for 5 days. Finally, to determine the potentiation of the antiviral effect of oseltamivir in the presence of ivy extract, PR8-infected mice received oral coadministration of ivy extract in the presence or absence of oseltamivir for 5 days. To confirm the anti-influenza activity of HSF in vivo, PR8-infected mice received 15 mg/kg HSF once daily by intraperitoneal administration for 5 days. Mice were weighed daily to assess body weight changes after PR8 virus infection.

### Viruses, cells, and reagents

PR8 virus was obtained from American Type Culture Collection (ATCC, Manassas, VA, USA). A549 cells purchased from ATCC (Rockville, MD, USA) were maintained in Dulbecco’s Modified Eagle’s Medium supplemented with 10% fetal bovine serum and 1% antibiotic-antimycotic solution, which were purchased from Gibco BRL (Invitrogen Life Technologies, Karlsruhe, Germany). L-(tosylamido-2-phenyl) ethyl chloromethyl ketone (TPCK)-modified trypsin was purchased from Thermo Scientific Pierce (Rockford, IL, USA). Both sulforhodamane B (SRB) and oseltamivir were purchased from Sigma-Aldrich (St. Louis, MO, USA). Tissue culture plates were purchased from BD Biosciences (San Jose, CA, USA). All other chemicals were of reagent grade.

### Fractionation and active compounds

For the fractionation of ivy extract, 30% EtOH extract of ivy leaf (*S*. *chinensis* Baill) was obtained from Ahngook Pharm (Seoul, Korea). The ivy extract (1.5 kg) was dissolved in 2 L of water and then partitioned with ethyl acetate and butanol, respectively. The butanol-soluble extract was evaporated under reduced pressure to yield 588.1 g of residue. The residue was subjected to DIAON HP-20 column chromatography (Sigma-Aldrich) eluted with gradient mixtures consisting of methanol/water (0:100 [fraction 1], 20:80 [fraction 2], 40:60 [fraction 3], 60:40 [fraction 4], 80:20 [fraction 5], 100:0 [fraction 6]; 2 × 500 mL each).

The major compounds found in ivy extract include hederacoside C, hederasaponin B (HSB), rutin, chlorogenic acid, and alpha-hedrin, which were obtained from Ahngook Pharm. HSF was isolated from ivy extract as described previously [16]. HSF was identified by ^1^H- and ^13^C-nuclear magnetic resonance (NMR) spectroscopy and mass spectrometry (MS) data analysis. Data were compared with published values [16,17]: ^1^H-NMR (600 MHz, pyridine-*d*_*5*_) *δ*: 6.21 (1H, d, *J* = 8.1 Hz, H-1′), 5.83 (1H, s, H-1′″), 5.39 (1H, s, H-12), 4.98 (1H, d, *J* = 7.8 Hz, H-1″), 1.68 (3H, d, *J* = 6.1 Hz, H-6′″), 1.33 (3H, s, CH_3_), 1.21 (3H, s, CH_3_), 1.06 (3H, s, CH_3_), 0.97 (3H, s, CH_3_), 0.90 (3H, s, CH_3_), 0.89 (3H, s, CH_3_), 0.87 (3H, s, CH_3_); ^13^C-NMR (125 MHz, pyridine-*d*_*5*_) *δ*: 176.57 (C-28), 144.12 (C-13), 122.81 (C-12), 104.71 (C-1″), 102.61 (C-1′″), 95.62 (C-1′), 85.00 (C-3), 78.61 (C-3′), 78.19 (C-4″), 77.93 (C-5′), 77.09 (C-5″), 76.43 (C-3″), 75.27 (C-2″), 73.87 (C-4′″), 73.80 (C-2′), 72.57 (C-3′″), 72.49 (C-2′″), 70.81 (C-4′), 70.26 (C-4′″), 69.16 (C-6′), 61.20 (C-6″), 56.20 (C-5), 47.91 (C-9), 47.02 (C-17), 46.20 (C-19), 42.08 (C-14), 41.63 (C-18), 39.83 (C-8), 38.83 (C-4), 38.65 (C-1), 37.12 (C-10), 33.99 (C-21), 33.13 (C-29), 33.02 (C-7), 32.50 (C-22), 30.74 (C-20), 28.71 (C-23), 28.24 (C-15), 26.14 (C-27), 24.85 (C-2), 23.74 (C-16), 23.69 (C-30), 23.34 (C-11), 18.67 (C-6), 18.46 (C-6′″), 17.45 (C-24), 17.09 (C-26), 15.56 (C-25); LC-ESI-MS (negative mode) *m/z*: 1005 [M-H]^+^, 535 [M-H-rhamnose– 2 x glucose]^+^. Purity of HSF was assessed by high-performance liquid chromatography as > 95% and the content of HSF in each fraction was measured by total ion current (TIC) chromatogram.

### Spectroscopy

Chromatography fractions were analyzed using an ACQUITY UPLC system (Waters Corporation, Milford, MA, USA) equipped with a binary solvent delivery manager, and a sample manger coupled to Micromass Q-TOF Premier mass spectrometer (Waters Corporation) equipped with an electrospray interface. Chromatographic separations were performed on a 2.1×100 mm, 1.7 μm ACQUITY BEH C18 chromatography column. The column temperature was maintained at 35°C, and the mobile phases A and B were water with 0.1% formic acid and acetonitrile with 0.1% formic acid, respectively. The gradient duration program was: 0 min, 20% B; 0 to 1 min, 20% B; 1 to 11 min, 20% to 65% B; 11 to 12 min, 65% to 98% B; 12 to 13.4 min, 98% B; 13.4 to 13.5 min 98% to 20% B; 13.5 to 15 min 20% B. The flow rate was 0.4 mL/min and the injection volume was 2 μL with partial loop mode. The mass spectrometer was operated in a negative ion mode with N_2_ used as the desolvation gas. The desolvation temperature was set to 350°C at a flow rate of 450 L/h and source temperature of 110°C. The capillary and cone voltages were set to 2,300 and 50 V, respectively. The data were collected for each sample from 100 to 1,500 Da with a 0.25-s scan time and a 0.01-s interscan delay over a 15-min analysis time. Leucine-enkephalin (m/z 554.2615) was used as a reference compound.

### In vitro antiviral activity assay

Antiviral activity was evaluated by the SRB method using cytopathic effect (CPE) reduction, as previously reported [18]. A549 cells were seeded in 96-well plates at a density of 2 x 10^4^ cells per well and incubated for 24 h. Diluted virus suspension containing 1 μg/mL TPCK-modified trypsin, and the selected concentration of compounds, were added to each well. Virus-infected cells without compounds treatment were used as viral controls, while non-infected cells without compounds treatment were used as cell controls. After incubation for 2 days, A549 cells were washed with PBS, and incubated with ice-cold 70% acetone for 30 min at -20°C. The acetone was removed and the plates were oven-dried for 30 min. Then, 0.4% (w/v) SRB in 1% acetic acid was added to each well for 30 min at room temperature. SRB was removed and the plates were washed with 1% acetic acid before oven-drying. After drying for 1 day, SRB was solubilized with 10 mM unbuffered Tris-based solution, and the absorbance was detected at 540 nm using a VersaMax Microplate Reader (Molecular Devices, Palo Alto, CA, USA) with a reference absorbance at 620 nm. The antiviral activity of each test compound in PR8-infected cells was calculated as a percentage of the corresponding untreated control. Oseltamivir and dimethyl sulfoxide were used as positive and negative controls, respectively.

### Histology and scoring

C57BL/6 mice (n = 5 mice/group) infected with PR8 virus (as described previously) were administered oral oseltamivir (5 mg/kg) and ivy extract (30 mg/kg) for 2 or 5 days. Lung tissue was rapidly excised from mice sacrificed 6 h after the final treatment. Lung tissue was washed with PBS and fixed in 4% formaldehyde for 1 h at 4°C. The tissue was dehydrated by gradual soaking in alcohol and xylene, and then embedded in paraffin. Paraffin-embedded specimens were cut into 10-μm sections, stained with hematoxylin and eosin (H&E), and viewed with a digital light microscope (Olympus, Tokyo, Japan). A pathologist performed a blind test of the sections under a light microscope and the levels of lung tissue destruction, epithelial cell layer damage, polymorphonuclear cell infiltration, and alveolitis were evaluated for scoring as previously described [19].

### Cytokine and chemokine analysis

The levels of chemokine (C-C motif) ligand 2 (CCL2), interleukin-6 (IL-6), and tumor necrosis factor-alpha (TNF-α) were measured by mouse ELISA Ready-SET-GO kit (eBioscience). The levels of chemokine (C-X-C motif) ligand 1 (CXCL1)/KC were measured by Duo Set Mouse ELISA Kit (R&D Systems), according to the manufacturer’s instructions.

### Flow cytometry

Cell were collected from bronchial alveolar lavage (BAL) fluid, and stained with the following antibodies: fluorescein isothiocyanate conjugated anti-CD11b, phycoerythrin-Cy7 tandem-conjugated anti-Ly6G, Allophycocyanin conjugated anti-CD11c, Phycoerythrin conjugated anti-F4/80. After staining, we analyzed those cells and found that there are several populations. Since CD11c^high^F4/80^high^ cells are known to as alveolar macrophages, we gated cells without them. We also get rid of cells which were not stained with any of antibodies, since they must be epithelial cells. Finally gated cells were analyzed for their expression of CD11b and Ly6G. All antibodies used for flow cytometry analysis were purchased from BD Biosciences (San Jose, CA). The cells were read by FACSVerse (BD Bioscience) and the data analyzed by BD FACSuite software application.

### Statistics

The Kaplan-Meier method was used to determine the statistical significance of differences in survival time. The Log-Rank test (Mantel-Cox) was performed using SPSS 12.0K for Windows to compare survival rate of PR8-infected mice after treatments. Student’s t-test was used to compare the differences between 2 groups, while one-way analysis of variance with Tukey-HSD post-hoc test was used to compare more than 2 groups. Values of p < 0.05 were considered to be significant.

## Results

### Ivy extract potentiates the antiviral activity of oseltamivir

**Fig 1:**
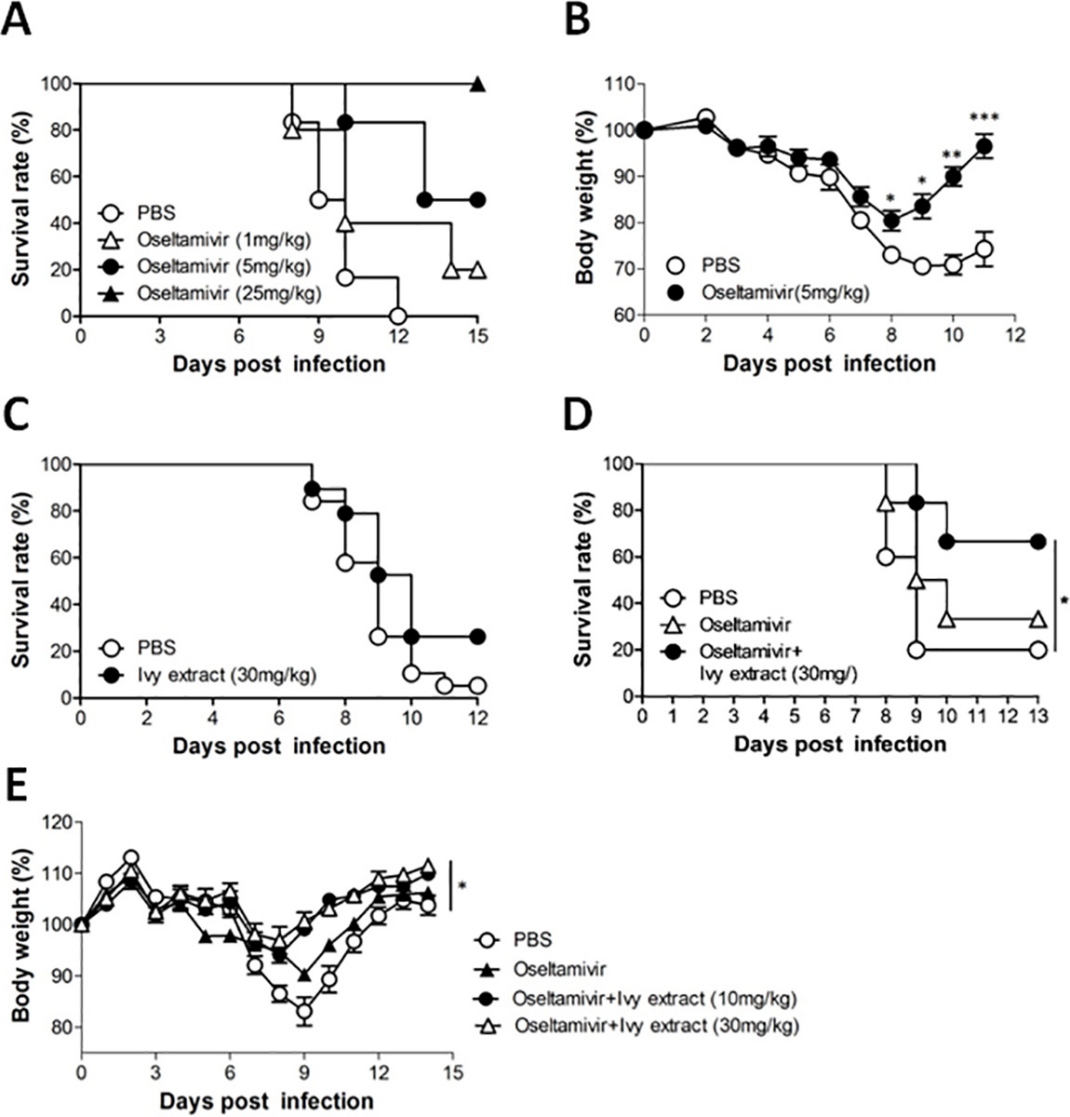
Determination of the effective dose of ivy extract in combination with oseltamivir. The survival rate (A) and body weight (B) of PR8-infected mice administered oseltamivir (*P<0.05; **P<0.01; ***P<0.001, two-tailed unpaired t-test). C: Survival rate of PR8-infected mice that received ivy extract or vehicle (PBS). D: Survival rate of PR8-infected mice that received oral coadministration of ivy extract and oseltamivir (*P<0.05, log-rank analysis of Mantel-Cox data) E: Body weight of PR8-infected mice that received oral coadministration of ivy extract and oseltamivir (*P<0.05, one-way ANOVA with Tukey’s post hoc test).

We adopted a novel strategy to screen extracts or chemical compounds eliciting antiviral activity under suboptimal oseltamivir against PR8 virus. Firstly, we determined the suboptimal dose of oseltamivir in PR8-infected mice. PR8-infected mice treated with 25 mg/kg of oseltamivir showed complete recovery from PR8 infection, while mice treated with 5 mg/kg showed 50% recovery (Fig 1A). Mice that survived the PR8 infection recovered their body weight within 8 days of infection; however, there were significant delays in recovery compared with mice receiving 5 mg/kg of oseltamivir treatment (Fig 1B). Thus, we decided to use 5 mg/kg oseltamivir for the combined treatment with candidate plant extracts in mice. We assumed that this dose of oseltamivir would enable us to assess the additive anti-influenza effects of plant extract in circumstances of incomplete removal of PR8 virus. Among the plant extracts tested during preliminary experiments, we found that 30% EtOH extract of ivy leaf (ivy extract) showed significant antiviral activity when combined with oseltamivir in vitro (data not shown). To confirm an anti-influenza virus effect of ivy extract in vivo, mice were intranasally infected with 5 x 10^3^ PFU of PR8 at day 0 and treated with 30 mg/kg of ivy extract for 5 days starting from day 2. Although the administration of ivy extract alone on PR8-infected mice increased the survival rate of mice, the effect was only marginal and statistically insignificant (Fig 1C).

We then assessed the potential anti-influenza effect of ivy extract in combination with oseltamivir. Coadministration of ivy extract with oseltamivir produced recovery from death in 60% of mice, whereas treatment with oseltamivir alone failed to rescue mice (Fig 1D). We also assessed the dose-dependent antivirus effect of ivy extract under suboptimal oseltamivir treatment on body weight in PR8-infected mice. We found that ivy extract administered at 10 mg/kg and 30 mg/kg protected mice from PR8 infection–mediated weight loss (Fig 1E). Collectively, these results suggested that coadministration of ivy extract with suboptimal treatment of oseltamivir assists mice to resist PR8 virus infection, and showed beneficial effect compared with oseltamivir treatment alone.

### Antiviral properties of ivy extract in vitro and in vivo

**Fig 2:**
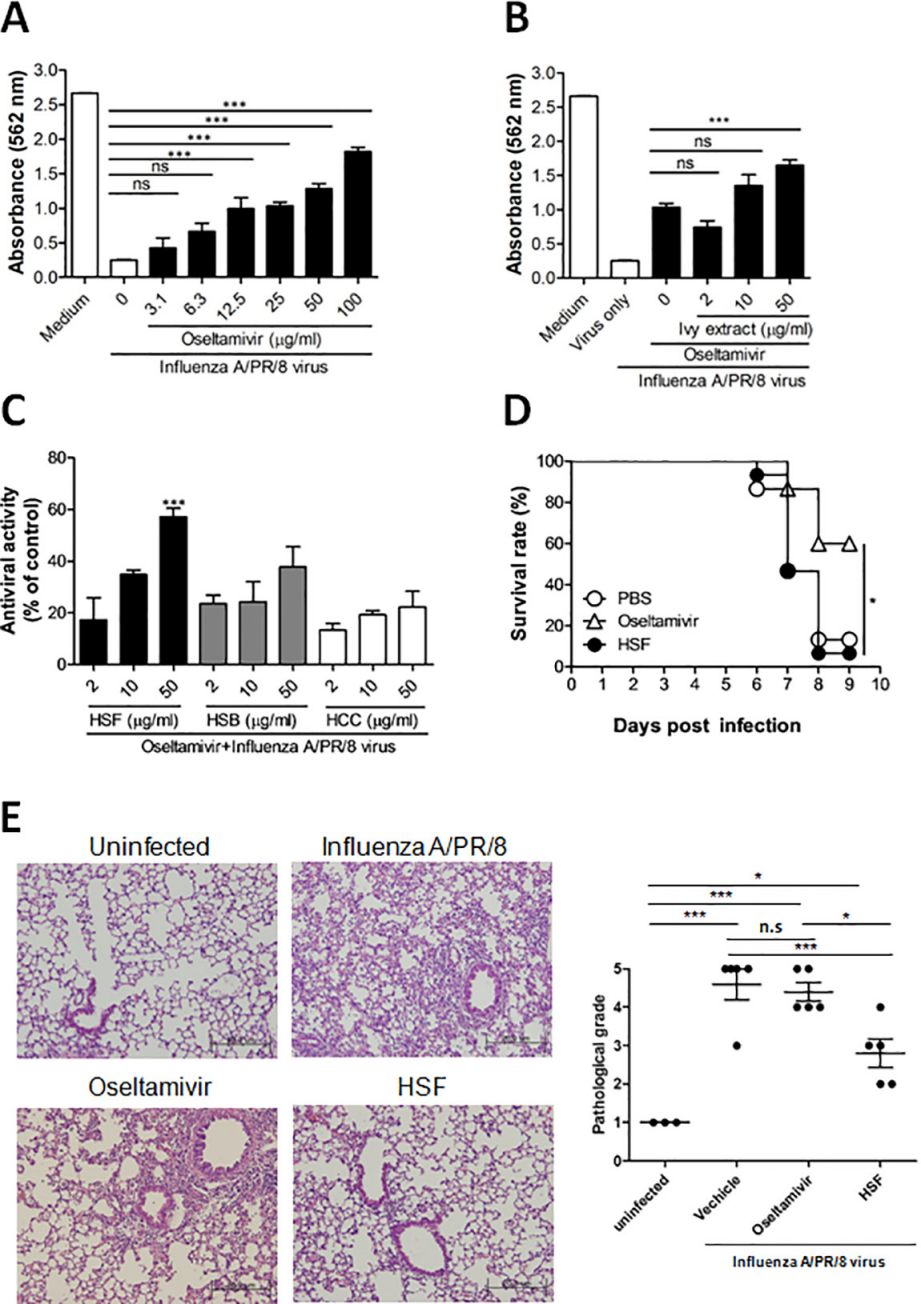
Antiviral activity of combined ivy extract and oseltamivir treatment against PR8 virus in vitro and in vivo. A: CPE reduction assay using SRB assay in A549 cells infected with PR8 virus were treated with oseltamivir for 48 h at the concentrations indicated. (***P<0.0001) B: Antiviral activity of ivy extract (at the concentrations indicated) combined with 25 μg/mL oseltamivir PR8-infected A549 cells. (***P<0.001) C: HSF, HSB, and hederacoside C were used in the presence of 25 μg/mL oseltamivir to identify its anti-PR8 virus activity in A549 cells. After 48 h of incubation, the antiviral activity was investigated by CPE reduction assay using SRB. (***P<0.001), and the antiviral activity was calculated based on the viability of virus infected cells as a percentage of the corresponding untreated control. Data are expressed as the mean ± SD of the percentage values obtained from 3 independent experiments carried out in triplicate. (***P<0.001). D: Survival of mice (n = 5/group) was monitored as depicted in Materials and Methods after treating PR8-infected mice with PBS, oseltamivir, or HSF (*P<0.05, log-rank analysis of Mantel-Cox data). E: Mice were infected with 5 x 10^3^ pfu/mouse of PR8, orally coadministered with HSF and/or oseltamivir from 2 days after PR8 infection for 5 days. Mice were sacrificed at 6h after final administration, and lung sections were prepared as described in Materials and Methods. Representative H&E stained samples of lung section were shown (left). Pathological grade of each mouse was evaluated (right). CPE, cytopathic effect; HSB, hederasaponin B; HSF, hederasaponin F; SRB, sulforhodamane B; H&E, hematoxylin and eosin. (*P<0.05) using one-way ANOVA with Tukey’s post hoc test.

To assess and verify in vitro antiviral activity of ivy extract in combination with oseltamivir, we first monitored the change in CPE induced by treatment with different concentrations of oseltamivir in PR8-infected A549 cells. The antiviral activity of oseltamivir against PR8 was determined using a 2-fold diluted concentration that ranged from 3.1 to 100 μg/mL. PR8 infection dramatically induced cell death in A549 cells, and treatment with oseltamivir increased the viability of virus-infected cells in a dose-dependent manner (Fig 2A). Based on our findings, we decided to use 25 μg/mL oseltamivir for the coadministration with ivy extract in vitro. Coadministration of 25 μg/mL oseltamivir and 50 μg/mL ivy extract exhibited significant antiviral activity against PR8 infection (Fig 2B).

To identify compounds responsible for the antiviral activity of ivy extract, we performed in vitro antiviral activity assays in A549 cells infected with PR8 virus using major compounds isolated from *H*. *helix* L. Among the tested compounds, only HSF showed significant anti-influenza virus activity in vitro when combined with oseltamivir (Fig 2C). The viability of PR8-infected A549 cells were measured after treatment of cells with HSF, HSB, or HCC, and the results are the relative resistance of A549 cells against PR8 infection-induced cell death. HSF in combination with 25 μg/mL oseltamivir demonstrated significant dose-dependent CPE against PR8 virus infection.

Subsequently, we assessed the antiviral effect of HSF *in vivo*. Although HSF alone did not render mice as significant protection against PR8 infection *in vivo* as oseltamivir did (Fig 2D), HSF treatment alone significantly attenuated pulmonary pathology induced by PR8 infection as assessed 7 days post-infection as compared with vehicle- and oseltamivir-treated mice (Fig 2E). Thus, there might be other factors critical for survival of mice after influenza infection than lung infiltration of inflammatory cells.

Together, these data demonstrate that HSF, which is contained in ivy extract, exhibits direct anti-influenza effects and might be responsible for the anti-influenza effect of combined ivy extract and suboptimal oseltamivir therapy.

### Combined ivy extract fraction and oseltamivir treatment alleviates PR8-induced lung inflammation

**Fig 3:**
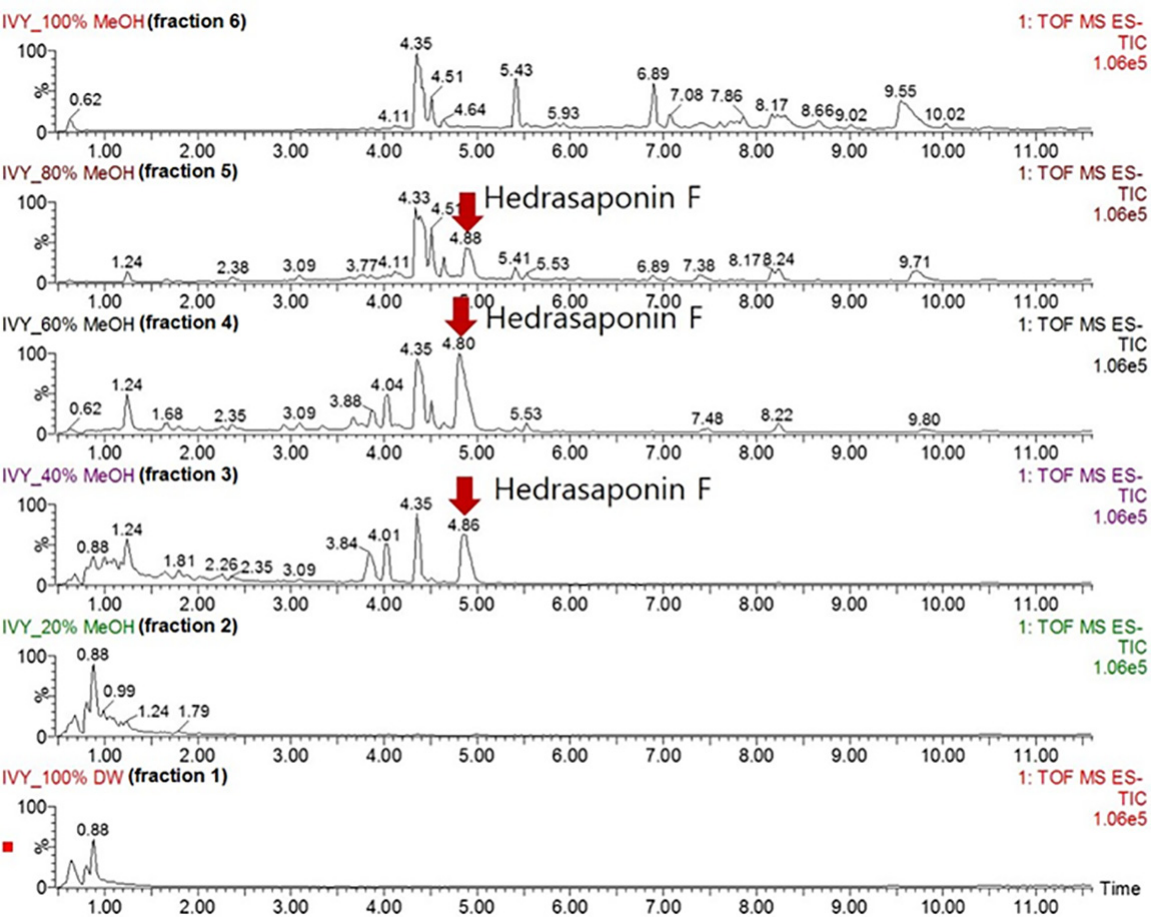
TIC chromatograms of chromatography fractions. The HSF content of each fraction analyzed using a chromatogram. HSF, hederasaponin F; TIC, total ion current.

To develop drug candidates for combined anti-influenza therapy with oseltamivir, we set out to find the fraction of ivy extract that predominantly contains HSF. Fig 3 shows the HSF content of each fraction. We confirmed that HSF was present in fractions 3, 4, and 5. Using fractionation of ivy extract with column chromatography and mass spectrometry analysis, we showed that the highest percentage of HSF was contained in the 60% MeOH fraction of ivy extract (fraction 4). Therefore, we selected fraction 4 for further experiments.

**Fig 4:**
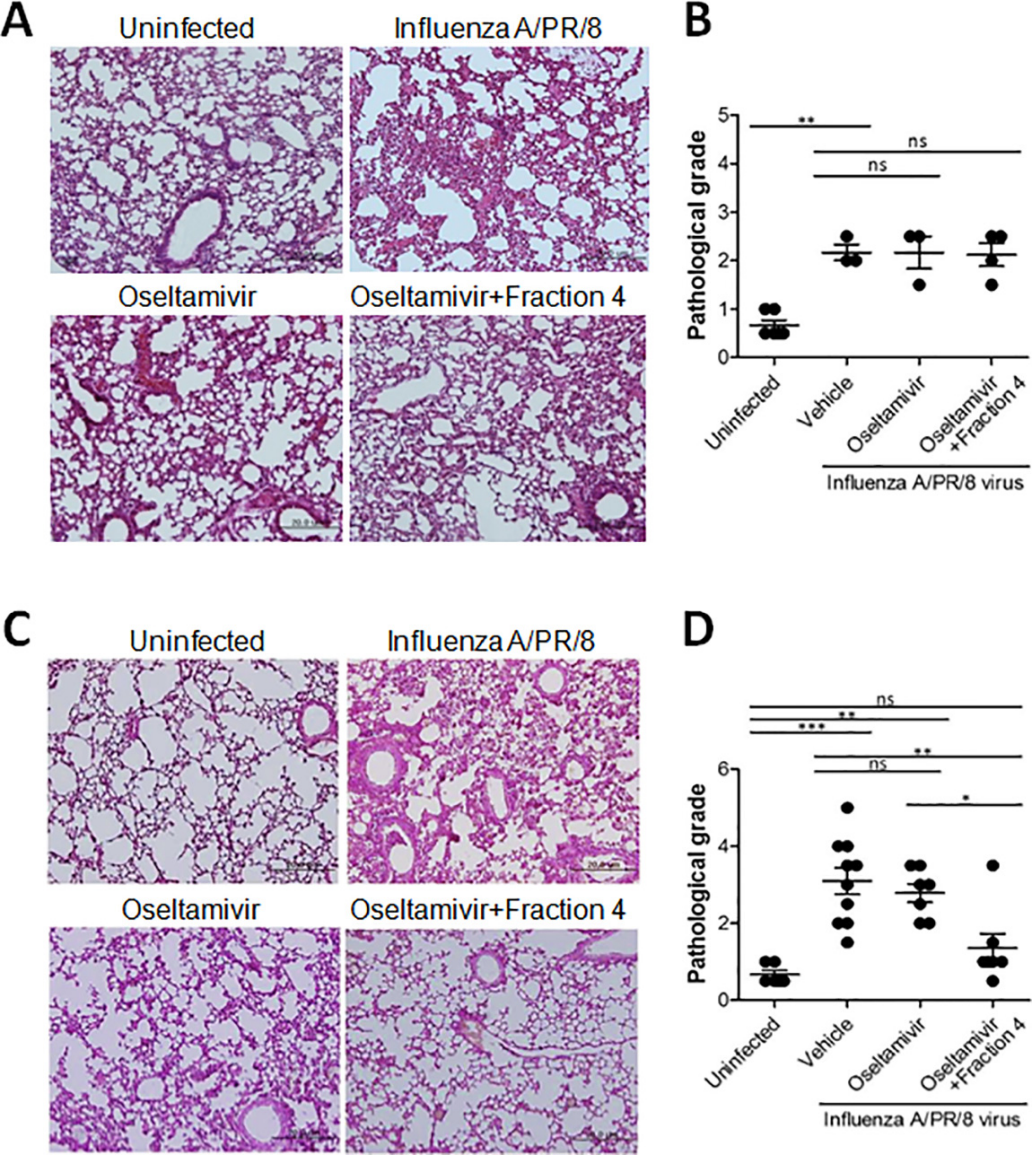
Anti-influenza virus activity of combined treatment with oseltamivir and fraction of ivy extract in mice. Representative H&E stained samples of lung section from uninfected or PR8-infected mice shown at 200x magnification. A and C: Infected mice were treated with oseltamivir alone or oseltamivir and fraction 4 of ivy extract (n = 4 per group) for 2 (A) or 5 (C) days. B and D: Pathological grade of mice that received oral drug administration for 2 (B) and 5 (D) days. *P<0.05;**p<0.01;***p<0.001; n.s., not significant. one-way ANOVA with Tukey’s post hoc test.

**Fig 5:**
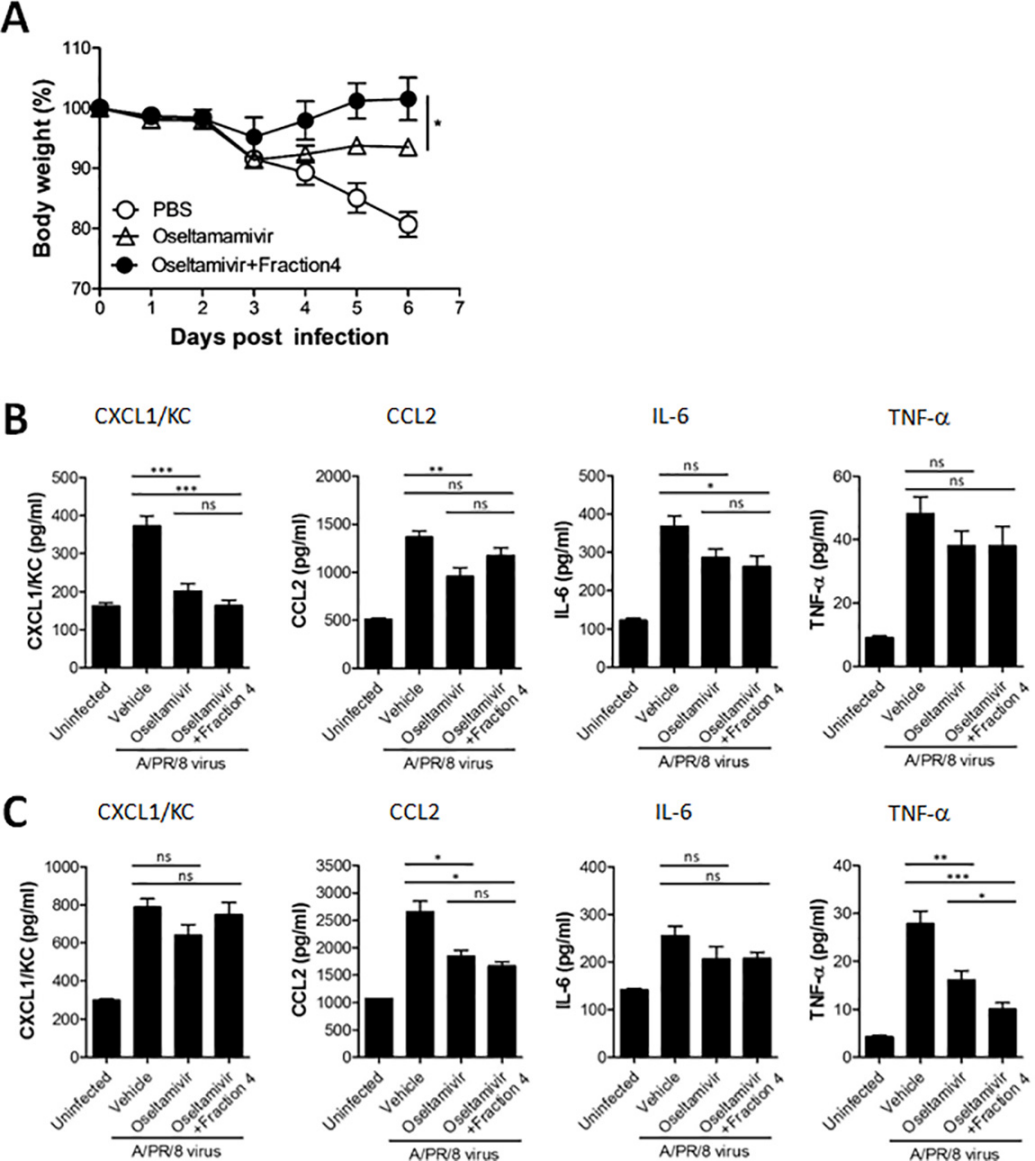
Cytokine production reduced by the combination of oseltamivir and fraction of ivy extract in mice. A: Body weight of PR8-infected mice administered with oseltamivir alone or coadministratered with oseltamivir and fraction 4 (*P<0.05, one-way ANOVA with Tukey’s post hoc test). B and C: Proinflammatory cytokines and chemokines measured from lung tissue of PR8-infected mice (n = 5 per group) treated with oseltamivir (5 mg/kg) in combination with fraction 4 of ivy extract (30 mg/kg) for 2 (B) and 5 (C) days. *P<0.05;**P<0.01;***P<0.001; n.s., not significant. one-way ANOVA with Tukey’s post hoc test.

We performed histological analysis on lung tissue obtained from uninfected mice and PR8-infected mice treated with 30 mg/kg fraction 4 and 5 mg/kg oseltamivir for 2 or 5 consecutive days. Lungs from uninfected C57BL/6 mice exhibited typical pulmonary tissue, whereas PR8-infected mice had inflammatory lesions characteristic of viral infection, including necrotizing bronchiolitis and interstitial pneumonia (Fig 4A and 4C). On the contrary, PR8-infected mice treated with a combination of oseltamivir and fraction 4 for 5 days had moderate inflammation with reduced necrosis, inflammatory cells infiltrates, and pulmonary edema compared with untreated PR8-infected mice 7 days post-infection (Fig 4C and 4D). We did not observe significant improvements in PR8-infected mice receiving combined fraction 4 and oseltamivir treatment for 2 consecutive days (Fig 4A and 4B). Although oseltamivir could successfully remove the virus from mice, our results indicate that oseltamivir treatment alone could not prevent pulmonary inflammation in PR8-infected mice. In contrast, combined treatment of PR8-infected mice with oseltamivir and the HSF-containing fraction 4 significantly reduced lung inflammation at post-infection day 7. In addition to the direct anti-influenza activity of HSF, combined oseltamivir and fraction 4 treatment might reduce pulmonary inflammation in the latter stage of PR8 virus infection. In addition, the disease symptom of PR8-infected mice was significantly alleviated by the combined treatment of fraction 4 with oseltamivir as compared with oseltamivir alone (Fig 5A), suggesting the complementary antiviral and anti-inflammatory activity of fraction 4 under suboptimal oseltamivir treatment.

### Combined ivy extract fraction and oseltamivir treatment alters pulmonary cytokines

Virus-induced chemokines and cytokines play a major role in recruiting leukocytes to the site of infection and activating innate immune responses to induce inflammation. Despite their protective roles, severe inflammation induced by a cytokine storm is associated with influenza-induced pulmonary pathology. We evaluated chemokine and cytokine production at the protein level in lung tissue from PR8-infected mice treated with oseltamivir in the presence or absence of fraction 4. Compared with PBS-treated control mice, intranasal infection of PR8 virus increased CXCL1, CCL2, IL-6, and TNF-α levels measured on post-infection days 4 and 7. Although oseltamivir significantly decreased CXCL1 and CCL2 levels in the lung of PR8-infected mice after two-times, daily treatment, coadministration of oseltamivir and fraction 4 did not induce significant changes in chemokine levels compared with oseltamivir treatment alone (Fig 5B). At post-infection day 7, the levels of pulmonary chemokine cytokine, especially CCL2, was significantly increased by PR8-infection and which was significantly decreased in groups treated with oseltamivir plus fraction 4. Interestingly, treatment of PR8-infected mice with five-times daily oseltamivir reduced TNF-α, and interestingly, combined oseltamivir and fraction 4 therapy more significantly decreased TNF-α compared with oseltamivir treatment alone in PR8-infected mice (Fig 5C). Although TNF-α reportedly plays an important function during virus infection, it also involved in pulmonary pathology after influenza infection. Collectively, our results suggest that fraction 4 decreases inflammatory cytokine levels, especially TNF-α, when used in combination with oseltamivir. Furthermore, the combined therapy enables PR8-infected mice to recover rapidly from severe pulmonary inflammation.

### Combined ivy extract fraction and oseltamivir treatment alters bronchoalveolar infiltrates

**Fig 6:**
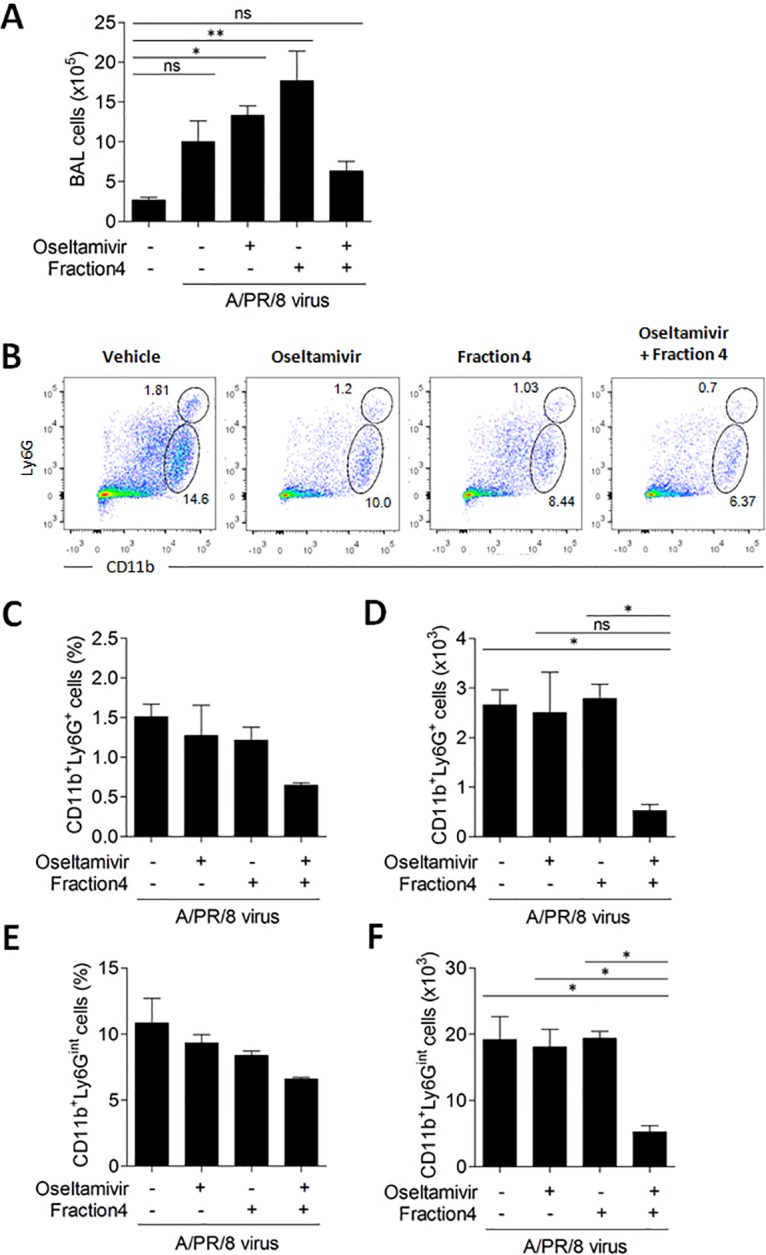
CD11b^+^Ly6G^+^ and Ly6G^int^ population reduced in mice treated with the combination of oseltamivir and fraction of ivy extract. A: Absolute number of cells in BALF from PR8-infected mice treated with oseltamivir alone or oseltamivir combined with fraction 4 of ivy extract for 5 days. B: Representative plots of CD11b and Ly6G levels assessed by flow cytometry. C and E: Percentages of cell populations in B were shown as a bar graph. D and F: The number of cells calculated were shown, *P<0.05; **P<0.01; ***P<0.001 (One-way ANOVA with Tukey’s post hoc test), BALF, bronchial alveolar lavage fluid.

We analyzed cellular infiltrates in BALF following oseltamivir treatment in the presence or absence of fraction 4 to determine whether the anti-inflammatory properties of fraction 4 reduce severe inflammation, directly target influenza, or cause death following PR8 infection. We did not detect significant changes in the total BAL cells number in PR8-infected mice treated with oseltamivir and fraction 4 (Fig 6A).

Recent studies suggest that myeloid cells consisting of neutrophilic cells and monocytic cells are involved in pulmonary inflammation induced by PR8 virus infection and might be associated with elevated levels of chemokines and cytokines in the lung [20]. Thus, we assessed cellular infiltration of neutrophils into BALF in PR8-infected mice treated with oseltamivir in the absence or presence of fraction 4. There were no significant changes in the percentages of CD11b^+^Ly6G^+^ cells in BAL (Fig 6B and 6C), and the number of CD11b^+^Ly6G^+^ cells having a granulocytic neutrophil phenotype was not significantly different in PR8-infected mice after oseltamivir treatment compared with vehicle-treated mice (Fig 6C). However, the number of CD11b^+^Ly6G^+^ cells was significantly reduced by the coadministration of oseltamivir with fraction 4 (Fig 6D). Similarly, although there was no significant change in the percentage CD11b^+^Ly6G^int^ cells having a monocytic phenotype infiltrated into the BALF of oseltamivir-treated PR8-infected mice compared with vehicle-treated mice (Fig 6B and 6E), the number of those cells was significantly decreased by the combined treatment of PR8-infected mice with oseltamivir and fraction 4 (Fig 6F). Overall, these results suggested that fraction 4 attenuated inflammatory cellular infiltrates in BALF induced by oseltamivir-treatment in PR8-infected mice.

## Discussion

The neuraminidase inhibitor oseltamivir is an antiviral drug licensed to prevent or slow the spread of influenza A and influenza B viruses [21], and has been recommended as standard therapy in the management of influenza viral infection. Although early administration of oseltamivir at high doses and increased duration of therapy showed significant anti-influenza virus effects in many preclinical studies [22], its efficacy is limited when its administration is delayed or because of drug resistance to the infected virus [23]. In the present study, we created a mouse model that reflects the clinical situation of influenza virus infection; we administered suboptimal doses of oral oseltamivir to mice 2 days after infection with a sublethal dose of PR8 virus. We found that ivy extract containing HSF showed significantly increased antiviral activity against the influenza virus when used in combination with oseltamivir. Furthermore, HSF and oseltamivir reduced pulmonary inflammation that occurs after influenza virus infection.

*H*. *helix* L. is commonly known as ivy or English ivy, and a member of the *Araliaceae* family. It is known to cause contact dermatitis [24]. Additionally, it has several biological activities, such as antibacterial [25], antihelmintic [26], leishmanicidic [27], in vitro antispasmodic [28], antifungal [29], and acute and chronic anti-inflammatory [30] properties. Ivy extract is believed to exhibit in vitro bronchodilatatory effects on cell cultures [28], and pharmaceutical manufacturers use ivy-based remedies to treat cough symptoms during the course of acute and chronic bronchitis. In our study, we reported that ivy extract significantly increased cell viability in PR8-infected A549 cells in combination with suboptimal dose of oseltamivir compared with oseltamivir treatment alone. We found that HSF contained in ivy extract showed significant anti-influenza activity, and might be responsible for the synergistic increase in anti-influenza virus activity by oseltamivir. Several major constituents of ivy extract reportedly exert moderate anti-influenza virus effects, and are dependent on inhibiting the formation of certain inflammatory mediators [13]. For example, α-hederin exhibits strong anti-inflammatory action.

We previously demonstrated that HSB is effective against EV71 [18]. HSB is a triterpenoid saponins [31] and this class of compound inhibits viral nucleotide synthesis against herpes simplex virus type-1 [32]. However, there have been no reports suggesting the antiviral activity of HSF.

Type 1 interferons and TNF-α are types of cytokines that play an important role in the early stage of virus infection. However, TNF-α is considered to be a detrimental proinflammatory cytokine [33,34], and TNF-α levels correlate with symptoms in human influenza virus infection [35]. This function of biological system is essential for triggering inflammation, host immune response, and recovery of tissue [36,37]. Although some studies reported that TNF-α had antiviral activity in lung epithelial cell [38,39], and TNF-α enhanced the expression of influenza virus–induced antiviral cytokines through Toll-like receptors/RIG-1-like receptors [40], the adverse effect of prolonged secretion of TNF-α under influenza viral infection needs to be evaluated. Here, we confirmed the level of TNF-α was significantly increased by PR8 infection, which was decreased in the lungs following coadministration of 60% MeOH fraction (fraction 4) of ivy extract containing HSF and oseltamivir. We presume that the enhanced recovery from PR8 virus infection after combined therapy was partially attributed to the reduced level of TNF-α that mediated the sustained pulmonary pathology postinfection.

Macrophages, dendritic cells, and natural killer cells are important for the initial control of virus [41] and certainly contribute to regulation of the adaptive immune responses during influenza virus infection. In particular, inflammatory monocytes with a Ly6C^hi^Ly6G^-^ phenotype are the dominant cells found in the lungs of mice during influenza virus infection [41]. Several recent studies also have described the involvement of inflammatory neutrophils in the respiratory tract during severe pulmonary inflammation following influenza virus infection. Both neutrophils and monocytes mediate the production of inflammatory cytokines and chemokines [42–44]. Interestingly, PR8 infection in mice increased the production of proinflammatory cytokines and chemokines, including CXCL1, CCL2, IL-6, and TNF-α, and the levels of CCL2 and TNF-α were decreased by oseltamivir treatment. In contrast, the infiltration of inflammatory cells was further increased by treating PR8-infected mice with oseltamivir. Although we could not analyze the functional characteristics of infiltrated cells in BALF after oseltamivir treatment, the levels of TNF-α and cellular infiltrates were significantly decreased by the coadministration of fraction 4 and oseltamivir compared with oseltamivir alone. This finding correlates with the elevated histologic attenuation of inflammation by fraction 4.

We proposed a novel anti-influenza virus therapy that can be used in combination with oseltamivir to prevent the PR8 virus. Furthermore, we determined the anti-influenza virus effect of using the 60% MeOH fraction of ivy extract that contains HSF in mice in combination with oseltamivir. Coadministration of ivy extract or 60% MeOH fraction of ivy extract with oseltamivir enables mice to recover more rapidly compared with suboptimal oseltamivir treatment. We also found that the antiviral activity of ivy extract in combination with oseltamivir might stem from the direct anti-influenza effect of HSF. In PR8-infected mice, the levels of pulmonary inflammatory cytokine, especially TNF-α, were significantly decreased in both of groups treated with oseltamivir or oseltamivir plus Fraction 4, which was more significantly decreased by the combination of oseltamivir and the Fraction 4 of ivy extract as compared with oseltamivir treatment alone. Interestingly, higher number of CD11b^+^Ly6^+^ and CD11b^+^Ly6^int^ cells infiltrated into the BALF of oseltamivir-treated mice after PR8 infection, which was significantly reduced by cotreatment with fraction 4. These results suggested that the effect of fraction 4 on the rapid recovery of PR8-infected mice might be attributable to an anti-inflammatory as well as anti-influenza effect of HSF. In conclusion, coadministration of ivy extract or fraction 4 containing HSF with oseltamivir enabled mice to overcome insufficient protection against PR8 virus infection under suboptimal treatment with oseltamivir. This was mediated by the antiviral activity of HSF and supported by anti-inflammatory activity of ivy extract.
